# Targeting GABARAPL1/HIF-2a axis to induce tumor cell apoptosis in nasopharyngeal carcinoma

**DOI:** 10.22038/IJBMS.2023.72952.15863

**Published:** 2024

**Authors:** Xiaopeng Huang, Liya Zhou, Jiawei Chen, Shuai Zhang

**Affiliations:** 1 Department of Radiation Oncology, Hainan General Hospital, Hainan Affiliated Hospital of Hainan Medical University, Haikou, Hainan Province 570311, People’s Republic of China; # These authors contributed equally to this work

**Keywords:** ATG4B, Autophagy, GABARAPL1, Hypoxia inducible factor, Nasopharyngeal carcinoma

## Abstract

**Objective(s):**

The primary gene mutations associated with nasopharyngeal carcinoma (NPC) are located within the phosphoinositide 3-kinase-mammalian target of rapamycin signaling pathways, which have inhibitory effects on autophagy. Compounds that target autophagy could potentially be used to treat NPC. However, autophagy-related molecular targets in NPC remain to be elucidated. We aimed to examine levels of autophagy-related genes, including autophagy-related 4B cysteine peptidase (ATG4B) and gamma-aminobutyric acid (GABA) type A receptor-associated protein-like 1 (GABARAPL1), in NPC cells and explored their potential role as novel targets for the treatment of NPC.

**Materials and Methods:**

The mRNA and protein expression of autophagy-related genes were detected in several NPC cells. Levels of GABARAPL1 were modified by either overexpression or knockdown, followed by examining downstream targets using RT-qPCR and western blotting. The role of GABARAPL1 in NPC proliferation and apoptosis was examined by flow cytometry. Furthermore, the role of GABARAPL1 was assessed in vivo using a nude mouse xenograft tumor model. The underlying mechanism by which GABARAPL1 regulated nasopharyngeal tumor growth was investigated.

**Results:**

Autophagy-related 4B cysteine peptidase (ATG4B), GABARAPL1, and Unc-51-like kinase 1 (ULK1) were significantly down-regulated in multiple NPC cell lines. Overexpression of GABARAPL1 up-regulated the expression of autophagy-related proteins, decreased the level of hypoxia-inducible factor (HIF)-2α, and induced apoptosis in NPC cells. Importantly, overexpression of GABARAPL1 slowed tumor growth. Western blotting showed that autophagy was activated, and HIF-2α was down-regulated in tumor tissues.

**Conclusion:**

HIF-2α, as a substrate for autophagic degradation, may play an interesting role during NPC progression.

## Introduction

Nasopharyngeal carcinoma (NPC) is a type of cancer with a subtle onset, high degree of local invasion, and common distant metastases. Despite comprehensive treatment with radiotherapy and chemotherapy, the five-year survival rate of NPC has remained around 70% (1). There are approximately 80,000 new cases and 50,000 deaths attributed to NPC in the world each year (1). Populations with high NPC incidence rates are concentrated in South China, Southeast Asia, and North Africa (2, 3). It is of great clinical significance to analyze the occurrence and development of NPC at the molecular level to discover novel disease mechanisms and improve patient survival rates (4-6).

It is known that the main gene mutations in NPC are located within phosphoinositide 3-kinase (PI3K)-mammalian target of rapamycin (mTOR) signaling pathways. These pathways have inhibitory effects on autophagy (7). Autophagy refers to the formation of autophagosomes from the double-membrane-wrapped cytoplasm where organelles and proteins are degraded within a cell and is important for the intracellular degradation of damaged organelles (7). Autophagy pathways have been extensively explored to study and develop therapeutic anti-cancer strategies (8). Classic autophagy-related (ATG) proteins can be divided into five categories: (i) Unc-51 like autophagy activating kinase 1 (ULK1) protein complex, (ii) Beclin-1/ phosphoinositide 3-kinase (PI3K) complex, (iii) ATG8/microtubule-associated protein 1A/1B light chain 3 (LC3) liposome binding system, (iv) ATG12-ATG5 protein binding system, and (v) ATG9 and its circulation system. The ATG8/LC3 lipid binding system includes two subclasses: gamma-aminobutyric acid (GABA) receptor-associated proteins (GABARAPs: GABARAP, GABARAPL1, GABARAPL2) and microtubule-associated protein 1A/1B light chain 3 proteins (MAP1LC3s: MAP1LC3A, MAP1LC3B, MAP1LC3C) (9, 10). 

Autophagy-related 4B cysteine peptidase (ATG4B) encodes a member of the autophagy protein family that plays an important role during autophagy (11). It has been reported to be up-regulated in numerous types of human malignancies, including chronic myeloid leukemia, breast cancer, and oral cancer (12-14). As an autophagy-related gene, GABARAPL1 is frequently down-regulated in a variety of tumor types, such as hepatocellular carcinoma and breast cancer (15, 16). Unc-51-like kinase 1 (ULK1) is also related to autophagy and can either promote or inhibit tumor growth (17).

Hypoxia-inducible factor (HIF)-2α can serve as a substrate for autophagic degradation, which affects tumor cell proliferation in NPC (18). When proteasome-mediated degradation is inactivated, autophagy-mediated protein degradation is compensatorily increased (18). Of note, compensatory autophagy is also inhibited following the accumulation of HIF-2α after Von Hippel–Lindau tumor suppressor (VHL) inactivation in NPC (18). This suggests the importance of understanding the regulatory pathways of autophagy and HIF-2α in NPC.

Based on the above background, it was hypothesized that autophagy plays a tumor suppressor role in NPC. The current study aimed to explore the effects of autophagy-related pathways in the development and progression of NPC. The investigation began by determining the levels of ATG4B, GABARAPL1, and LC3 in NPC. Next, potential anti-tumor effects were explored. Lastly, the study was expanded to possible downstream targets of autophagy, thereby providing novel effector molecules that could be used to target and treat NPC. 

## Materials and Methods

***Cell lines and culture***

Two normal cell lines (human bronchial epithelial [HBE] cells and human immortalized esophageal epithelial clone-strong [NE1] cells) and three human NPC cell lines (6-10B, NE2, and HNE1) were purchased from the BeNa Culture Collection. Cells were cultured in RPMI 1640 medium with fetal bovine serum (10%), penicillin (100 U/ml), and streptomycin (100 µg/ml) in a humidified incubator at 37 °C with 5% CO_2_. Media was changed every 2–3 days. 

***Transmission electron microscope***

Cultured cells were fixed in 2.5% Karnovsky’s solution for at least two hours and then post-fixed for 1–2 hr in 1% osmium tetroxide buffered with 0.1M cacodylate buffer. Dehydration of the slice followed a series of alcohol and acetone washes at 4 °C. Next, infiltration was to expose the tissue to one or more mixtures of 100% acetone and embedding medium. After all samples were embedded, the molds were cured in a 37 °C oven overnight followed by 12 he in a 45 °C oven and then 48 hr in a 60 °C oven. After the molds were cured, the specimens were rough-trimmed with EMUC7. The resin block is placed in a vice and trimmed by shaving it into a trapezoid with dimensions approximately 70 nm thick. Grids are stained with 2% uranyl acetate-lead citrate double staining and then imaged by TEM HT7800 (80KV).

***RNA extraction and quantitative polymerase chain reaction (qPCR)***

RNA extraction was performed using the Total RNA Extraction Kit (R1200, Solarbio Life Sciences, Beijing, China). cDNA was synthesized using Hifair® III 1st Strand cDNA Synthesis SuperMix kit (11141ES60, Yeasen, Shanghai, China). qRT-PCR was performed with Hieff® qPCR SYBR Green Master Mix (11201ES08, Yeasen, Shanghai, China). Data was analyzed based on the 2-ΔΔCt method (19). Primer sequences are listed in Table 1. 

***Western blotting***

Cell lysis was performed using radioimmunoprecipitation assay buffer (P0013B, Beyotime, Shanghai, China). Protein concentration was measured with a bicinchoninic acid kit (PC0020, Solarbio, Beijing, China). Protein samples were separated by sodium dodecyl-sulfate polyacrylamide gel electrophoresis and then transferred to a polyvinylidene fluoride membrane. After blocking with 5% fat-free milk (232100, BD), the membrane was probed with primary antibodies at 4 °C overnight. After the membrane was washed, it was incubated with the appropriate secondary antibody at room temperature for 1 hr. The antibodies used are listed in Table 2. An enhanced chemiluminescence reagent (Beyotime, Shanghai, China) was used to expose protein bands on an imaging system (Tanon 5200). Protein levels were quantified using Image J. 

***Transfection***

Cells were seeded onto a 6-well plate (3x105 cells/well). After 24 hr, transfection was performed in Opti-MEM medium with either GABARAPL1 overexpression plasmid or GABARAPL1 knockdown plasmid, for 20 min. Cells were incubated for another 48 hr and then used for subsequent analysis. The media was changed every 24 hr. 

***Cell apoptosis and proliferation***

After transfection, cells were harvested for either apoptosis or proliferation analysis using flow cytometry. Apoptosis was tested with annexin V and propidium iodide (PI) staining. Proliferation was assessed using 5-ethynyl-2’-deoxyuridine (EdU) and Hoechst 33342 staining.

***Animal model***

All animal experimental protocols were approved by the Animal Care Committee of Hainan General Hospital, and Hainan Affiliated Hospital of Hainan Medical University. Four-week-old BALB/c nude mice were purchased from SPF Biotechnology Co. Ltd. (Beijing). Mice were randomized into two groups, and were subcutaneously injected in the right armpit region with 2.0 × 106 6-10B cells with or without GABRAPL1 overexpression. Tumor volume was monitored with calipers and was calculated based on the formula: V=L*W*W/2, in which L was the longest dimension and W was the shortest dimension perpendicular to L. At the end of the experiment, the mice were euthanized. Tumor tissues were isolated and weighed. 

***H*** ***&*** ***E staining***

Mouse tumors were placed in labeled and numbered embedding cassettes, followed by dehydration and embedding in 70%, 80%, 90%, 95%, and 100% alcohol followed by xylene and paraffin. Tissue slicing was then dewaxed and dehydrated. After dehydration, the slices were stained with hematoxylin for 1 min and then eosin for 1 min. Finally, the slides were sealed with neutral Balsam. Slides were imaged under a light microscope Nikon Ci-S (Nikon, Japan) with Nikon DS-U3 software.

***Statistical analysis***

Statistical analysis and data plotting were performed using GraphPad Prism. Comparisons were made according to unpaired two-tailed t-tests. All experiments performed consisted of three independent replicates. Data are presented as mean ± standard deviation (SD). Ordinary one-way ANOVA, multiple comparisons were used and a *P*-value<0.05 was considered statistically significant. 

## Results

***Decreased levels of ATG4B and GABARAPL1 in NPC***

**Figure 1:**
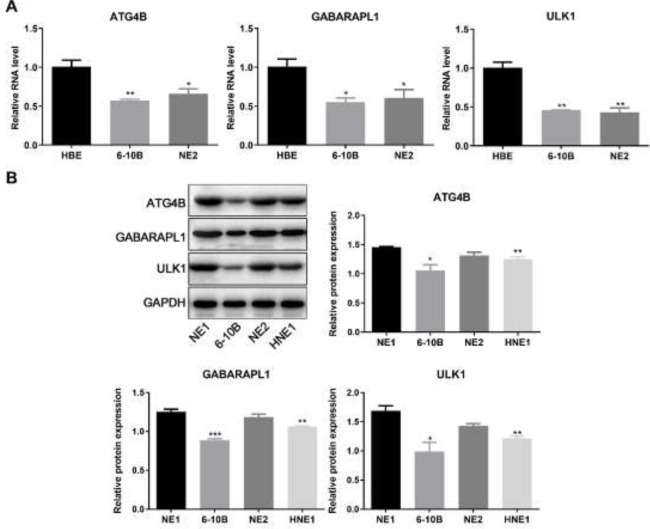
Levels of ATG4B and GABARAPL1 in NPC cell lines

To explore the role of autophagy in NPC, the levels of ATG4B, GABARAPL1, and related autophagic genes in NPC cells were first assessed via quantification of mRNA levels by qPCR and protein levels by western blotting (Figure 1). It was found that the mRNA levels of ATG4B, GABARAPL1, and ULK1 were decreased in NPC cells compared to control HBE cells (Figure 1A). Consistently, the protein levels of ATG4B, GABARAPL1, and ULK1 were lower in NPC cells compared to control NE1 cells (Figure 1B). The above findings indicate that the decreased levels of ATG4B and GABARAPL1 could play an important role in NPC.

***GABARAPL1 overexpression enhances apoptosis in NPC***

**Figure 2:**
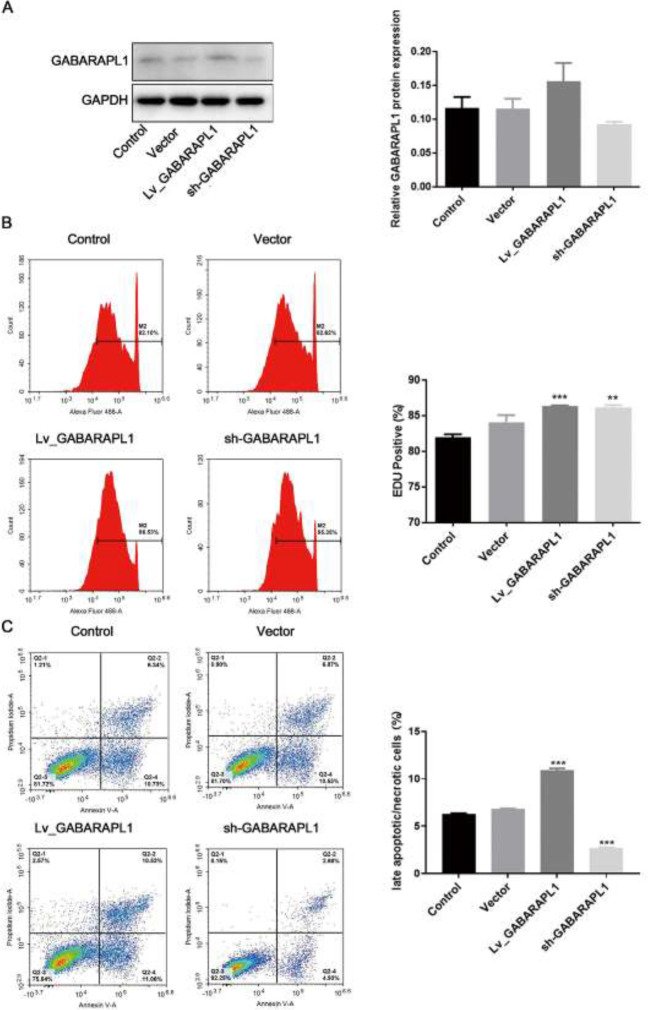
Effects of GABARAPL1 overexpression/knockdown in NPC 6-10B cell line

To explore the potential of GABARAPL1 as a target for the treatment of NPC, the effects of GABARAPL1 on rates of apoptosis and proliferation in the NPC cell line 6-10B were tested. Cell lines were constructed with either the overexpression or knockdown of GABARAPL1. The overexpression or knockdown of GABARAPL1 was confirmed by western blotting (Figure 2A). EdU staining data revealed that overexpression of GABARAPL1 enhanced the proliferation of 6-10B cells (Figure 2B). Next, cells were cultured for 24 hr before the apoptosis assessment by annexin V/PI staining. As shown in Figure 2C, overexpression of GABARAPL1 enhanced apoptosis in 6-10B cells, while knockdown of GABARAPL1 decreased rates of apoptosis in 6-10B cells (Figure 2C). This confirmed the role of GABARAPL1 in promoting the apoptosis of NPC cells. 

**Figure 3:**
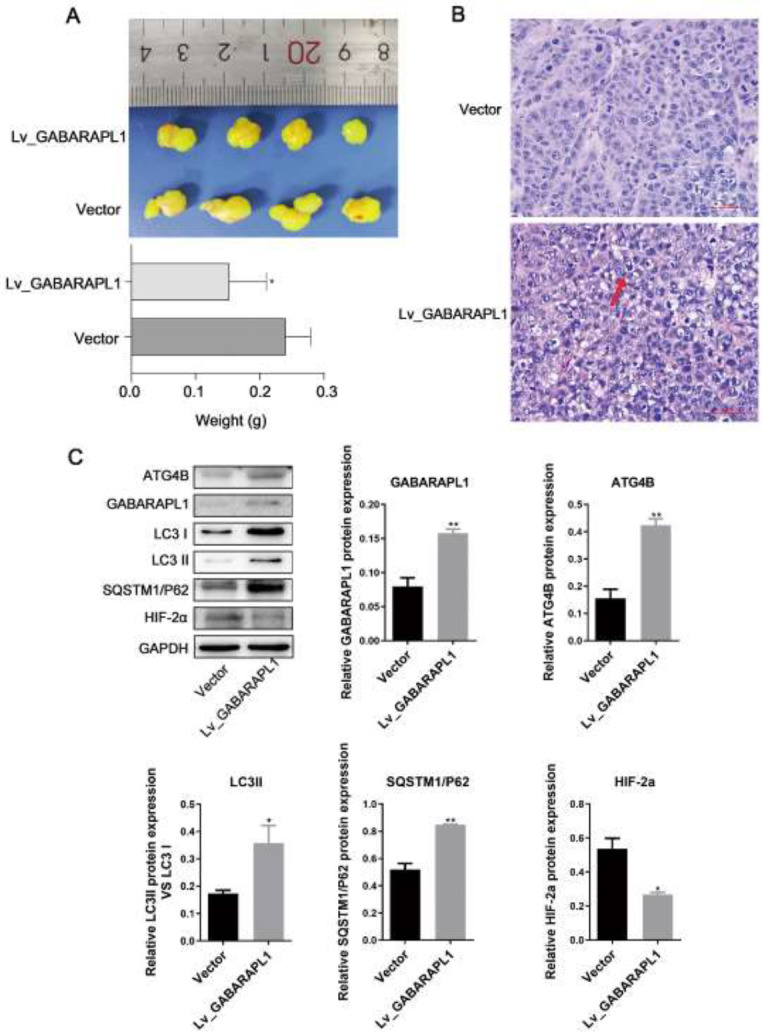
Effect of GABARAPL1 on HIF-2α

To explore if the effects of GABARAPL1 on 6-10B cells could be translated to an in vivo model, 6-10B cells with or without the overexpression of GABARAPL1 were subcutaneously inoculated into nude mice. As shown in Figure 3A, the overexpression of GABARAPL1 speeded up tumor growth in nude mice. H&E staining confirmed the more severe tumorigenesis in the GABARAPL1 overexpression group (Figure 3B). At the endpoint, the levels of GABARAPL1 in the tumor tissues from mice were confirmed by western blot, which confirmed overexpression of GABARAPL1 in mouse tumor tissues (Figure 3C). Interestingly, the levels of ATG4B, LC3II/LC3I, and SQSTM1/P62 were all up-regulated in mouse tumor tissues that overexpressed GABARAPL1 (Figure 3C), suggesting an up-regulation of autophagy in the tumor. More importantly, the levels of HIF-2α were decreased (Figure 3C), indicating a possible mechanism by which up-regulating autophagy led to the digestion of the HIF-2α protein. 

***GABARAPL1 overexpression leads to a decrease in HIF-2α levels***

**Figure 4:**
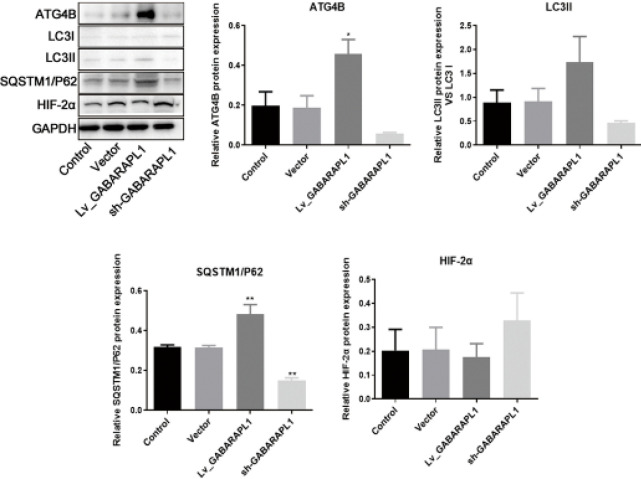
Effects of GABARAPL1 overexpression or knockdown on related genes in NPC 6-10B cell line

To explore the axis by which GABARAPL1 affected the progression of NPC, a 6-10B cell line with overexpression of GABARAPL1 was constructed (Figure 2A). Interestingly, upon GABARAPL1 overexpression, the protein levels of other genes in the axis including ATG4B, LC3II/ LC3I, and SQSTM1/P62 were all up-regulated (Figure 4). Of note, when GABARAPL1 levels were low, HIF-2α levels were high (Figure 4). This suggests that GABARAPL1 possibly exerts an anti-tumor effect in NPC via HIF-2α.

**Figure 5:**
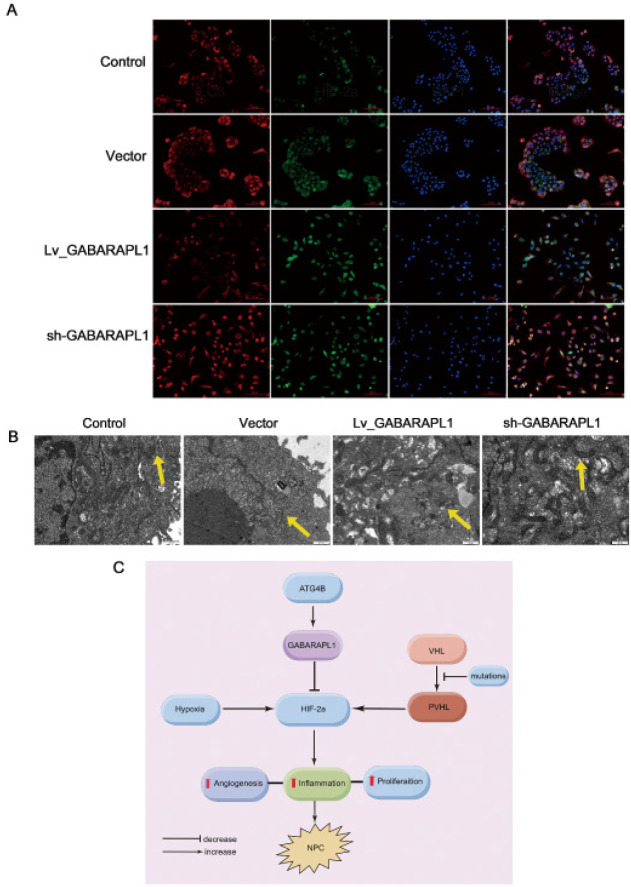
Effect of GABARAPL1 on HIF-2a via autophagy

To investigate whether GABARAPL1 regulates HIF-2a via an autophagy-related pathway, immunofluorescence staining was performed on 6-10B cells. As shown in Figure 5A, the results confirmed the colocalization of HIF-2α with LC3. The autophagy activities in these cells were further examined using a transmission electron microscope. The formation of autophagosomes within the cells was evaluated (Figure 5B). Compared to control cells 6-10B and GABARAPL1 knockdown cells, overexpression of GABARAPL1 evidently promoted the autophagy of HIF-2α (Figure 5B).

In NPC, HIF-2α acts as an oncogene. It can be speculated that the ATG8/LC3 liposome binding system mediated by ATG4B in autophagy can be targeted to induce the decrease of autophagic degradation product HIF-2α and reduce the aggregation of HIF-2α in NPC (Figure 5C). This can be used as a new NPC treatment strategy.

**Table 1:** Primer sequence for autophagy-related genes and internal reference

**Gene**	**Primer sequence**	
GAPDH	F	TCAAGAAGGTGGTGAAGCAGG
	R	GCGTCAAAGGTGGAGGAGTG
ATG4B, mouse	F	TCAGGAAGTGGGTGTGTGGGAAA
	R	GGCAATTCTCAGCAAGGCAAGGA
LC3B, mouse	F	TTCAGGTTCACAAAACCCGC
	R	TCTCACACAGCCCGTTTACC
LC3A, mouse	F	GCCTTCTTCCTGCTGGTGAACC
	R	TCCTCGTCTTTCTCCTGCTCGTAG
HIF2α, mouse	F	CCGAACTGACCAGATATGACTGTGAG
	R	AGTCTGCCAGGTAAGTCCATCTTGTA
GABARAPL1, mouse	F	CGGAAGAGAATCCACCTGAGACCT
	R	TCCAGTATTGTGCAACCAGAACCATT
GABARAPL1, human	F	TCTCCATCTGGCTCTCCTCTACCT
	R	TGGTCCTCCTTGTACTGGAACTTCAT
p62, mouse	F	AGTCGGATAACTGTTCAGGAGGAGAT
	R	AGCCAGCCGCCTTCATCAGA

GAPDH: glyceraldehyde 3-phosphate dehydrogenase; ATG4B: autophagy-related 4B cysteine peptidase; LC3A: microtubule-associated protein 1 light chain 3 alpha; LC3B, microtubule-associated protein 1 light chain 3 beta; HIF-2α: hypoxia-inducible factor 2 alpha; GABARAPL1: gamma-aminobutyric acid (GABA) type A receptor-associated protein-like 1

**Table 2:** Primer sequence for autophagy-related proteins and internal reference

**Antibody target**	**Dilution factor**	**Cat # and source**
ATG4B	1:1000	10482-1-AP, Proteintech
GABARAPL1	1:1000	66575-1-Ig, Proteintech
SQSTM1/P62	1:1000	AF5384, Affinity
HIF-2α	1:500	NB100-902HIF2, Novus
LC3I/II	1:500	SAB3500350, Sigma-Aldrich
GAPDH	1:5000	10494-1-AP, Proteintech

ATG4B: autophagy-related 4B cysteine peptidase; GABARAPL1: gamma-aminobutyric acid (GABA) type A receptor-associated protein-like 1; SQSTM1/P62: HIF-2α, hypoxia-inducible factor 2 alpha; LC3I/II: microtubule-associated protein 1 light chain 3 I/II; GAPDH: glyceraldehyde 3-phosphate dehydrogenase

A. Relative mRNA levels of ATG4B, GABARAPL1, and ULK1 in different NPC cell lines (HBE, 6-10B, and NE2). B. Western blot analysis of ATG4B, GABARAPL1, and ULK1 expression in different NPC cell lines (NE1, 6-10B, NE2, and HNE1). Grayscale analysis of western blot indicates protein expression levels relative to GAPDH. Biological triplicates were performed. Data are presented as mean ± SD. Ordinary one-way ANOVA and multiple comparisons were used for analysis. ***P<0.001, **P<0.01, *P<0.05.

ATG4B: autophagy-related 4B cysteine peptidase; GABARAPL1: gamma-aminobutyric acid (GABA) type A receptor-associated protein-like 1; NPC: nasopharyngeal carcinoma; ULK1: Unc-51 like autophagy activating kinase 1; GAPDH: glyceraldehyde 3-phosphate dehydrogenase; SD: standard deviation; ANOVA: analysis of variance

A. Overexpression or knockdown of GABARAPL1 was confirmed by Western blotting after 24 hr transfection. Grayscale analysis of western blot indicates the protein expression levels relative to GAPDH. B. EdU staining flow cytometry of NPC cells after overexpression or knockdown of GABARAPL1. The percentage of EdU-positive cells is shown. C. Apoptosis flow cytometry of NPC cells after overexpression or knockdown of GABARAPL1. Annexin V-FITC/PI was used. The percentage of late apoptotic cells is shown. Biological triplicates were performed. Ordinary one-way ANOVA and multiple comparisons were used for analysis. Data are presented as mean ± SD. ***P<0.001, **P<0.01, *P<0.05. Control, cells without interventions. Vector, cells transfected with empty vector. Lv_GABARAPL1, cells with overexpressed GABARAPL1. Sh_GABARAPL1, cells with knockdown of GABARAPL1.

GABARAPL1: gamma-aminobutyric acid (GABA) type A receptor-associated protein-like 1; NPC: nasopharyngeal carcinoma; GAPDH: glyceraldehyde 3-phosphate dehydrogenase; EdU: 5-ethynyl-2’-deoxyuridine; FITC: fluorescein isothiocyanate; PI: propidium iodide; ANOVA: analysis of variance; SD: standard deviation

A. NPC 6-10B cell line-derived tumor growth in BALB/c nude mice. 6-10B cells with stable transfection of empty vector or GABARAPL1 were injected into nude mice (four mice/group). The tumor size and the tumor weight at the endpoint (15 days after cell injection) are shown. B. Representative images for H&E staining of tumor tissues from mice. C. Western blot analysis of GABARAPL1-related targets in the tumor tissues from mice at the endpoint. Grayscale analysis of western blot indicates the protein expression levels relative to GAPDH. Biological triplicates were performed. Data are presented as mean ± SD. Ordinary one-way ANOVA and multiple comparisons were used. ***P<0.001, **P<0.01, *P<0.05. Vector, tumors with empty vector. Lv_GABARAPL1, cells with overexpressed GABARAPL1.

GABARAPL1: gamma-aminobutyric acid (GABA) type A receptor-associated protein-like 1; HIF-2α: hypoxia-inducible factor 2 alpha; NPC: nasopharyngeal carcinoma; H&E: hematoxylin, and eosin; GAPDH: glyceraldehyde 3-phosphate dehydrogenase; SD: standard deviation; ANOVA: analysis of variance

Representative image of western blotting and quantitative analysis of protein levels on ATG4B, LC3, P62, HIF-2α after overexpression or knockdown of GABARAPL1. Biological triplicates were performed. Data are presented as mean ± SD. One-way ANOVA and multiple comparisons were used for analysis. ***P<0.001, **P<0.01, *P<0.05. Control, cells without interventions. Vector, cells transfected with empty vector. Lv_GABARAPL1, cells with overexpressed GABARAPL1. Sh_GABARAPL1, cells with knockdown of GABARAPL1.

GABARAPL1: gamma-aminobutyric acid (GABA) type A receptor-associated protein-like 1; NPC: nasopharyngeal carcinoma; ATG4B: autophagy-related 4B cysteine peptidase; LC3: microtubule-associated protein 1 light chain 3; HIF-2α: hypoxia-inducible factor 2 alpha; SD: standard deviation; ANOVA: analysis of variance

A. Representative immunofluorescence staining on 6-10B cells after knockdown or overexpressed GABARAPL1. Colocalization of HIF-2α and LC3 is shown. Scale bar: 100 µm. Red indicates LC3. Green indicates HIF-2α. Blueindicates DAPI. Yellow indicates colocalization of HIF-2α and LC3. B. Representative images of autophagosome formation after knockdown or overexpressed GABARAPL1 under a transmission electron microscope. Yellow arrows indicate autophagosomes. Compared to the control, empty vector, and GABARAPL1 knockdown cells, the autophagosome formation is increased in cells with overexpression of GABARAPL1. Scale bar: 500 µm. C. Mechanistic schematics of regulatory pathways. The cascade regulations of ATG4B, GABARAPL1, and HIF-2a in NPC cells are shown. Control, cells without interventions. Vector, cells transfected with empty vector. Lv_GABARAPL1, cells with overexpressed GABARAPL1. Sh_GABARAPL1, cells with knockdown of GABARAPL1.

GABARAPL1: gamma-aminobutyric acid (GABA) type A receptor-associated protein-like 1; HIF-2α: hypoxia-inducible factor 2 alpha; LC3: microtubule-associated protein 1 light chain 3; DAPI: 4’,6-diamidino-2-phenylindole; ATG4B: autophagy-related 4B cysteine peptidase; NPC: nasopharyngeal carcinoma

## Discussion

Autophagy can degrade waste components in cells into nutrient components necessary for cell survival. When autophagy is overactivated, cells can also be stimulated by this “self-digesting” degradation pathway, ultimately leading to increased apoptosis (8). Thus, autophagy has been studied as a potential target for the treatment of NPC. Studies have found that decreased levels of autophagy and decreased mRNA expression levels of autophagic markers ULK1 and Beclin-1 can indicate better prognosis for patients with NPC (20-23). The autophagy-related gene ATG4B, which plays a crucial role in the autophagy process by delipidation of LC3, is also inversely associated with prognosis in NPC patients (24). Though the prognosis significance of the autophagy gene GABARAPL1 in NPC patients has not been studied yet, low expression of GABARAPL1 has been demonstrated to be associated with poor prognosis in hepatocellular carcinoma and lymph node-positive breast cancer (15, 16). Moreover, the interplay between autophagy and other signaling pathways such as mTOR, TGF-β, and HIF pathways in cancer development has been under investigation. The mTOR protein kinase can regulate autophagy through inhibitory phosphorylation of ULK1 (25) while TGF-β can increase the expression of numerous autophagy-related genes such as Beclin-1 to activate autophagy in certain breast cancer and hepatocellular carcinoma cell lines (26). MicroRNA-185 inhibits NPC by negatively regulating the TGF-β1/mTOR axis, thus enhancing autophagy (27). The role of HIF-1/2 in tumor progression through autophagy has been well elucidated (28, 29). Autophagic degradation of HIF2α has been demonstrated to suppress renal tumorigenesis (30). In line with the above results, the current study demonstrated that autophagy played a tumor-suppressive role in NPC. When GABARAPL1 was overexpressed, the autophagosome formation indicator LC3 was induced, HIF-2α was decreased, NPC cell apoptosis was enhanced, and tumor cell growth was inhibited in the cultured NPC cells and the mouse xenograft model.

As an intracellular degradation pathway, autophagy can inhibit tumorigenesis by degrading oncogenic proteins. In NPC, HIF-2α, an oncogene transcription factor, serves as a substrate for autophagic degradation, suggesting an important mechanism by which autophagy manipulates the occurrence and metastasis of NPC. If the key regulators in autophagy can be targeted to activate and increase autophagic degradation, while reducing the accumulation of HIF-2α in NPC caused by pVHL inactivation, it can be used as a novel NPC treatment approach.

Under normoxic conditions, the α subunit of HIF can be hydroxylated by three different prolyl hydroxylases, which in turn are ubiquitinated by the E3 ubiquitin ligase complex and subsequently degraded. Under hypoxic conditions, the hypoxia prevents the occurrence of hydroxylation, and the α subunit of HIF is transferred to the nucleus where it binds to the constitutively expressed β subunit, thereby inducing gene expression of components containing hypoxia response elements (HRE) (31, 32). The presence of HRE has been shown to regulate the expression of vascular endothelial growth factor, platelet-derived growth factor, epidermal growth factor receptor, and transforming growth factor-α (33, 34).

In NPC, the anti-cancer HIF-1α is not usually expressed, while HIF-2α, a transcriptional regulator with oncogenic effects, regulates downstream processes involved in angiogenesis, glucose metabolism, and tumor growth target genes (35, 36). In a hypoxic microenvironment, VHL gene inactivation and other proteasomal degradation pathway factors all lead to the accumulation of intracellular HIF-2α, which plays a crucial role in the development of NPC (37-39). Additional studies have found that the use of HIF-2α antagonists can inhibit the proliferation rate of various NPC cell lines and reduce the tumor diameter in mouse models (40, 41). HIF-2α can serve as a substrate for autophagic degradation and ultimately affects tumor cell proliferation in NPC (7). In the future, strategies to specifically target HIF-2α could potentially be developed into a novel method to treat NPC.

## Conclusion

In summary, it was demonstrated that the GABARAPL1/HIF-2α axis regulates the progression of NPC* in vitro *and *in vivo*. Overexpression of GABARAPL1 led to increased apoptosis in NPC cells and slowed tumor growth in nude mice. This could shed light on a novel therapeutic strategy targeting GAPARAPL1 in NPC. Further studies on up-regulating the autophagic degradation of HIF-2α in NPC may open new avenues for the treatment of NPC.

## Authors’ Contributions

All authors made substantial contributions to the conception and design, acquisition of data, or analysis and interpretation of data; took part in drafting the article or revising it critically for important intellectual content; agreed to submit to the current journal; gave final approval of the version to be published; and agree to be accountable for all aspects of the work.

## Funding

This study was supported by the Hainan Provincial Natural Science Foundation of China, (No. 820MS129). The authors thank all research staff for their contributions to this project.

## Availability of Data and Materials

The datasets generated and analyzed during the present study are available from the corresponding author upon reasonable request. 

## Ethics Approval and Consent to Participate

This study was approved by the ethics committee of Hainan General Hospital, Hainan Affiliated Hospital of Hainan Medical University (NO:2023-31). All applicable international, national, and/or institutional guidelines for the care and use of animals were followed.

## Conflicts of Interest

The authors declare that they have no conflicts of interest.
